# Auditory training during development mitigates a hearing loss-induced perceptual deficit

**DOI:** 10.3389/fnsys.2014.00049

**Published:** 2014-04-04

**Authors:** Ramanjot Kang, Emma C. Sarro, Dan H. Sanes

**Affiliations:** ^1^Center for Neural Science, New York UniversityNew York, NY, USA; ^2^Department of Biology, New York UniversityNew York, NY, USA

**Keywords:** conductive hearing loss, plasticity, auditory training, frequency modulation, development, perceptual learning

## Abstract

Sensory experience during early development can shape the central nervous system and this is thought to influence adult perceptual skills. In the auditory system, early induction of conductive hearing loss (CHL) leads to deficits in central auditory coding properties in adult animals, and this is accompanied by diminished perceptual thresholds. In contrast, a brief regimen of auditory training during development can enhance the perceptual skills of animals when tested in adulthood. Here, we asked whether a brief period of training during development could compensate for the perceptual deficits displayed by adult animals reared with CHL. Juvenile gerbils with CHL, and age-matched controls, were trained on a frequency modulation (FM) detection task for 4 or 10 days. The performance of each group was subsequently assessed in adulthood, and compared to adults with normal hearing (NH) or adults raised with CHL that did not receive juvenile training. We show that as juveniles, both CHL and NH animals display similar FM detection thresholds that are not immediately impacted by the perceptual training. However, as adults, detection thresholds and psychometric function slopes of these animals were significantly improved. Importantly, CHL adults with juvenile training displayed thresholds that approached NH adults. Additionally, we found that hearing impaired animals trained for 10 days displayed adult thresholds closer to untrained adults than those trained for 4 days. Thus, a relatively brief period of auditory training may compensate for the deleterious impact of hearing deprivation on auditory perception on the trained task.

## Introduction

The developmental sensory environment can initiate life long modifications to central nervous system computations and has been demonstrated for a broad range of sensory systems (for review see (visual) Hubel, 1978; Wiesel, 1982; Hooks and Chen, 2007; (somatosensory) Feldman and Brecht, 2005; (auditory) Keuroghlian and Knudsen, 2007; Sanes and Bao, 2009). In the auditory system, these neural deficits are closely associated with impaired perceptual skills. For example, developmental auditory deprivation leads to diminished behavioral performance on frequency discrimination, amplitude modulation detection, and sound localization (Clements and Kelly, 1978; Kerr et al., 1979; Knudsen et al., 1984; Moore et al., 1999; Rosen et al., 2012). Consistent with this, developmental hearing loss in humans can lead to persistent deficiencies in sound localization and signal detection, as well as impairments in the acquisition of speech and language (Hall and Grose, 1994; Wilmington et al., 1994; Hall et al., 1995; Kidd et al., 2002; Halliday and Bishop, 2005, 2006). Even transient periods of conductive hearing loss (CHL), due to chronic otitis media with effusion, may cause perceptual deficits (Whitton and Polley, 2011).

In contrast to the detrimental impact of developmental hearing loss, a brief period of auditory training during development can enhance performance on the trained task when animals are tested in adulthood (Sarro and Sanes, 2011). The long-term effect, as assessed in adulthood, is similar to the impact of adult perceptual training (Wright et al., 1997; Wright and Fitzgerald, 2001; Wright and Sabin, 2007). However, the short term effect of training during development can be surprisingly limited (Sarro and Sanes, 2010; Huyck and Wright, 2011). These findings suggest that developmental auditory training could counteract the long-term perceptual deficits induced by early sound deprivation. Here, we asked whether the diminished performance skills of adult animals reared with CHL could be rescued with a brief period of auditory perceptual training during juvenile development.

In the present study, we examined the performance of gerbils on a frequency modulation (FM) detection task. Adults reared with CHL display much poorer performance on this task than adults reared with normal hearing (NH; Buran et al., 2014). Therefore, we provided both CHL and NH juveniles with a brief period of auditory training on the FM detection task, and reassessed their detection thresholds in adulthood. Juvenile training permitted animals with hearing loss to display superior FM detection thresholds and decreased variance (as measured with psychometric function slope) as compared to CHL animals without juvenile training. These results suggest that training induces a long-term compensation for the perceptual deficits on the trained task caused by early hearing loss.

## Materials and Methods

### Animals

Gerbil (*Meriones unguiculatus*) pups were weaned from commercial breeding pairs (Charles River) at postnatal days (P) 23–30. Males and females were caged separately and maintained in a 12 h light/dark cycle. All procedures related to the maintenance and uses of animals were in accordance with the “Institutional Animal & Use Committee Handbook” and approved by the University Animal Welfare Committee (UAWC) at New York University.

### Developmental hearing loss

Bilateral CHL was induced via surgical removal of a middle ear bone, the malleus, prior to the onset of hearing at P10. At postnatal day 10 (P10), pups were anesthetized with the halogenated ethyl methyl ether, methoxyflurane. Anesthetic induction occurred within 10 min and produced complete elimination of responses to nociceptive stimuli. CHL was induced by tympanic membrane puncture and malleus extirpation (Tucci et al., 1999). A postauricular skin incision was made, and the tympanic membrane was visualized and punctured with a forceps. The malleus was then removed through this opening. The postauricular wound was closed with cyanoacrylate glue, and procedure repeated on the other side. After surgery, animals were warmed on a heating pad and returned to the litter when respiration and motor activity had recovered. The age of surgery was chosen based on the finding that anteroventral cochlear nucleus cell number is unaffected by cochlear ablation after P9 in gerbils (Tierney and Moore, 1997). This manipulation induces an attenuation of ≈55 dB at 4 kHz, as assessed by auditory brainstem response (Tucci et al., 1999; Rosen et al., 2012), but behavioral measures indicate an attenuation of ≈40 dB at 4 kHz (Buran et al., 2014). We did not use sham controls for this study, however previous work from our lab has published findings that show similar effects of CHL when compared to sham controls or non-sham controls (Takesian et al., 2012; Kotak et al., 2013), demonstrating the neurophysiological effects of CHL were not due to the anesthesia or surgery procedures.

### Experimental groups

FM depth detection thresholds were obtained from gerbils as both juveniles and adults. Data from adults reared with NH, and adults reared with CHL were collected previously for a study on the critical period of vulnerability to hearing loss (Buran et al., 2014). Both of these groups received procedural training and 10 days of perceptual training (described below) on an FM detection task from ∼P70–P90. The new data collected in this study was obtained from the following groups: (1) Juvenile trained NH animals (*n* = 11 (2f, 9m)) received procedural training and 4 or 10 days of perceptual training on an FM detection task beginning on ∼P23, the earliest age at which animals could be weaned and placed on controlled water access; (2) Juvenile trained CHL gerbils (*n* = 13 (7f, 6m)) also received procedural training and 4 or 10 days of perceptual training on an FM detection task beginning at ∼P23. As adults (∼P70–P90), after the age at which gerbils reach sexual maturity (Field and Sibold, 1999) these animals were retested on the FM detection task; (3) Adult trained NH gerbils (*n* = 6 (6m)) received procedural training and 10 days of perceptual training on an FM detection task beginning at ∼P70. As older adults (∼P120–150) these animals were retested on the FM detection task. These animals were not exposed to handling or the testing context prior to adult testing; (4) An additional group (*n* = 6 (2f, 4m)) of adults reared with NH were obtained, and added to the data obtained previously and re-shown here (Buran et al., 2014). These animals were not exposed to handling or the testing context prior to adult testing.

### Behavioral testing apparatus

Gerbils were placed in a testing cage of approximated 1 ft^2^ that was housed within a sound isolation booth (Gretch-Ken Industries), and observed from a separate room via a closed circuit monitor. When the animal contacted both a metal footplate and lick spout, they completed a circuit that initiated water delivery via a syringe pump (Yale Apparatus). A personal computer, connected to a digital interface (Tucker-Davis Technologies, TDT RZ6), generated the acoustic stimuli, timed the water delivery (0.3 ml/min), and controlled a small current that was delivered through the metal lick spout. Auditory stimuli were delivered via a calibrated tweeter (KEF Electronics) positioned 1 m in front of the test cage at 0° elevation. Sound level was calibrated with a spectrum analyzer (Bruel and Kjaer 3550) via a 1/4″ free-field condenser microphone positioned at the head location when in contact with the lick spout.

### Procedural training

All training used a conditioned avoidance Go-Nogo procedure to measure detection of FM stimuli (Heffner and Heffner, 1995; Kelly et al., 2006; Sarro and Sanes, 2010, 2011). Animals were placed on controlled water access and, upon introduction to the experimental cage, learned to obtain water from the metal lick spout. This training occurred in the presence of a continuous unmodulated 4 kHz tone. Sound level was set to 45 dB SPL for NH animals, and 95 dB SPL for animals with CHL to compensate for the elevated thresholds. These values were identical to those used in a published report on FM detection (Buran et al., 2014). Additionally, we previously found that sensation level was not correlated with FM detection threshold (Buran et al., 2014). Animals were then trained to withdraw from the spout when a FM stimulus was presented. To train the withdrawal response, a low AC current (0.5–1.0 mA, 300 ms; Lafayette Instruments) was delivered through the lick spout immediately after each FM stimulus. Since animals display between-subject variability in pain sensitivity (Mogil, 1999; Wasner and Brock, 2008; Nielsen et al., 2009) the strength of the shock was adjusted for each animal to reliably produce withdrawal from the spout, but not so great as to dissuade an animal from approaching the spout on subsequent trials (Sarro and Sanes, 2010). To train animals on the procedure, go trials (4 kHz center frequency; 5 Hz modulation rate; 500 Hz modulation depth) were presented until performance reached a criterion of ≥70% correct over 10 consecutive trials. All animals received procedural training to establish criterion performance when they were first introduced to the task as juveniles, and again when they were reintroduced to the task for assessment in adulthood.

### Perceptual training and assessment of FM detection thresholds

Once this criterion was reached, we tested animals on a range of at least 5 FM depths within each session, presented in descending order from largest to smallest. On each subsequent day of perceptual training, an animal’s performance on the previous day determined the range of depths that were presented; these depths always bracketed the previous day’s detection threshold. This protocol was used for all treatment groups. For juvenile perceptual training, animals were trained for either 4 or 10 days. This choice was chosen to remain consistent with the number of juvenile training days used in a prior study (Sarro and Sanes, 2011). Subsequently, we found that adults approach their best detection thresholds on the FM detection task when using at least 10 days of detection training (Buran et al., 2014). Adult animals were tested until their performance stabilized (i.e., did not improve for 3 consecutive days).

### Trial structure

Each trial was 1000 ms long, either containing the FM stimulus (go trials) or not containing a modulation of the 4 kHz center frequency (nogo trials). To determine if the animal detected the FM stimulus, contact with the spout was monitored during the final 100 ms of each go trial. A contact time of <50 ms was scored as a correct response (i.e., a hit), and a contact time of >50 ms was scored as a miss. For nogo trials, a contact time of <50 ms was scored as a false alarm, and a contact time of >50 ms was scored as a correct rejection. Go trials always occurred after a block of 3–5 nogo trials, randomized to avoid temporal conditioning.

### Data analysis

**Figure 1:**
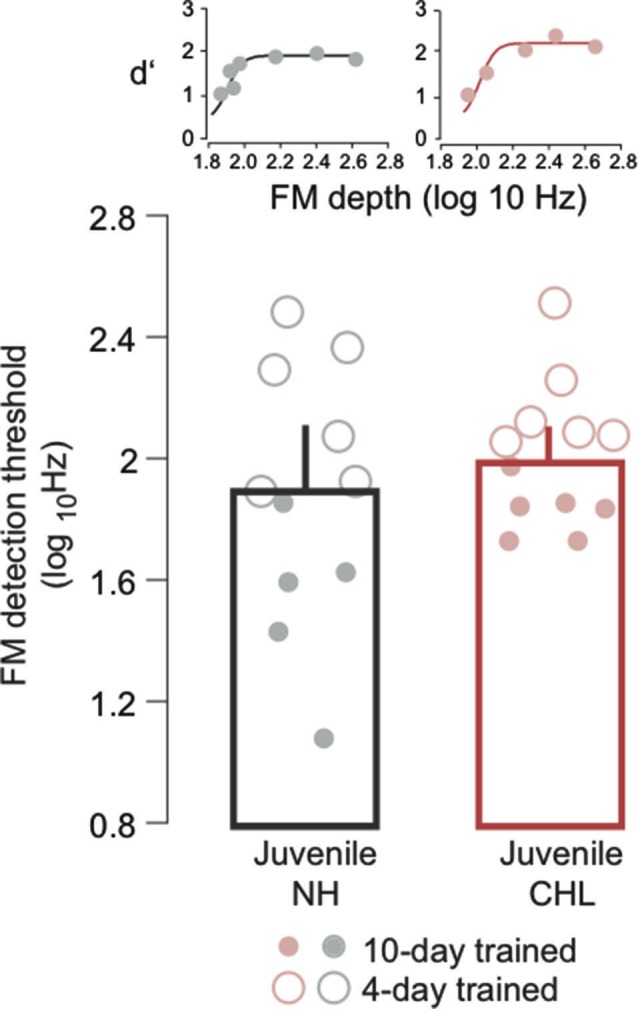
**Normal hearing (NH) and conductive hearing loss (CHL) juveniles display similar frequency modulation (FM) detection thresholds**. Average FM detection thresholds (log_10_Hz; mean ± SEM) are plotted for NH juveniles (black bar, gray circles) and CHL juveniles (red bar; light red circles). Data points from individual animals are the average of the best 3 days of performance. Open circles indicate 4 days of perceptual training, and closed circles indicate 10 days of perceptual training. Insets: Example psychometric function fits from one NH juvenile and one CHL juvenile, taken from a single day of testing.

**Figure 2:**
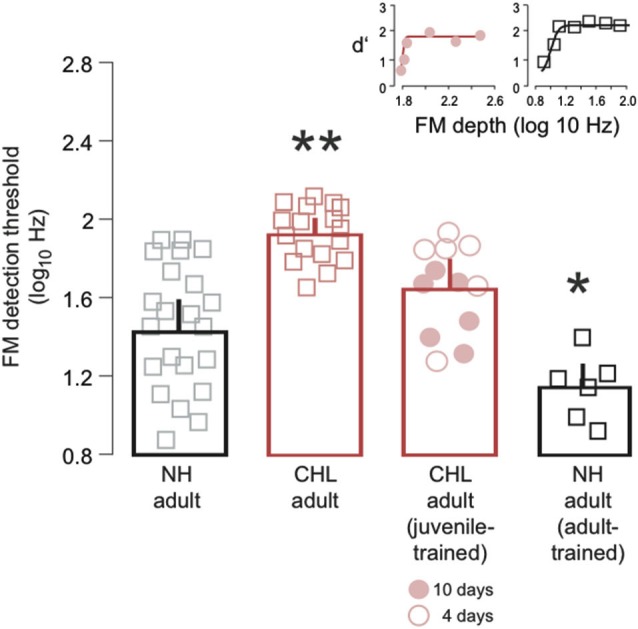
**Training on FM detection as a juvenile improved detection thresholds assessed in adulthood**. Average FM detection thresholds (log_10_Hz; mean ± SEM) are plotted for NH adult (black bar, gray squares), CHL adult (red bar; red squares), CHL adult animals trained on the FM task as juveniles (red bar; red circles), and NH adult animals trained on the FM task as adults and retested at a later time (black bar, black squares). For the juvenile trained animals, open circles indicate 4 days of perceptual training, and closed circles indicate 10 days of perceptual training. Data points from individual animals are the average of the best 3 days of performance. Significant differences for between-group comparisons are presented in the text and indicated here by asterisks (** significantly greater than all other groups; * significantly smaller than all other groups). Insets: Example psychometric function fits from CHL adult animals trained on the FM task as juveniles (red circles, red line) and the NH adult animals trained on the FM task as adults and retested at a later time (black bar, black squares), taken from a single day of testing. Examples from the same set of NH adults and CHL adults are provided in a previous published report (Buran et al., 2014).

Behavioral sensitivity, *d*′ = *z*(false alarm) − *z*(hit), was obtained for *z*-scores that corresponded to the right-tail *p*-values (Swets, 1973), and was calculated for each FM depth. Only sessions in which an animal performed a minimum of 5 trials per stimulus value were included in the analysis. Performance functions from sessions consisting of at least five presentations of five different depths were fitted using the open-source package psignifit as described in Buran et al. (2014). To ensure fits were of sufficient quality we discarded fits where the deviance of the fit to the original dataset exceeded the 95th percentile of the deviance of the fit to 1,000 simulated datasets (see Fründ et al., 2011 for details). Threshold was defined as the FM depth at which performance reached a *d*′ = 1, and slope was also calculated at a *d*′ of 1, as obtained from fitted psychometric functions (see examples shown in Figures 1, 2). Average threshold for each treatment group was determined by averaging the threshold for the best 3 days of testing for each animal.

Statistical tests were first performed to determine whether the dependent variable was normally distributed for each treatment group (NH, CHL, juvenile-trained CHL), using a Shapiro-Wilk normality test. We found two departures from normality (slope of psychometric function for NH adults, *p* = 0.02 and Juvenile-trained CHL adults, *p* = 0.007) and used Levene’s test for equal variance (using the median value as an estimate of each group’s center) since it is more robust when samples deviate from a normal distribution. Two of the dependent variables displayed unequal variance (Adult FM thresholds, *df* = 2, *F* = 4.6, *p* = 0.02; and slope of psychometric function, *df* = 2, *F* = 33.6, *p* < 0.001). For all multiple comparison tests, we used the non-parametric Kruskal-Wallis test followed by pairwise comparisons using a two-sided Wilcoxon test with Holm-corrected *p*-values. For normally distributed depended variables (Juvenile FM detection thresholds), we used a one-way ANOVA as a way to compare the groups.

## Results

### FM detection ability for NH and CHL gerbils is similar during development

To address whether developmental CHL influenced perception during the juvenile period, we obtained FM detection thresholds from NH and CHL animals at P23–P40. Figure 1 plots the mean FM detection thresholds, and the performance of individual animals, with days of training indicated by the symbol type. An ANOVA reported a main effect of training duration (*F* = 35.0, *df* = 1, *p* < 0.0001), but there was neither an effect of hearing status (*F* = 3.3, *df* = 1, *p* = 0.08) nor an interaction between hearing status and training duration (*F* = 2.8, *df* = 1, *p* = 0.11). Therefore, it appears that CHL does not immediately diminish FM detection thresholds.

### Adult FM detection is improved by juvenile training

We have previously shown that developmental CHL leads to degraded FM detection thresholds in adulthood (Buran et al., 2014). Here, we tested whether training on the FM detection task during juvenile development could rescue this perceptual skill in adulthood. NH and CHL animals were trained on the FM detection task from P23–P40, and retested as adults. Figure 2 illustrates a main effect of treatment group on FM detection thresholds (Kruskal-Wallis: χ^2^ = 31.03, *df* = 3, *p* < 0.0001). The average FM detection threshold for NH adults (1.44 ± 0.05 log_10_Hz) was significantly lower than for CHL adults (1.93 ± 0.06 log_10_Hz) (Wilcoxon test: χ^2^ = 19.8, *df* = 1, *p* < 0.0001). Notably, the average FM detection threshold for juvenile trained CHL-reared animals (1.64 ± 0.07 log_10_Hz) was significantly better than CHL adults (Wilcoxon test: χ^2^ = 10.73, *df* = 1, *p* < 0.001), and only slightly worse than the detection thresholds of NH animals (Wilcoxon test: χ^2^ = 3.4, *df* = 1, *p* = 0.07). However, the CHL animals that had been trained for 10 days (Figure 2, closed red circles; 1.54 ± 0.09 log_10_Hz) generally displayed better detection thresholds than those animals trained for only 4 days (Figure 2, open circles; 1.74 ± 0.09 log_10_Hz). This suggests that developmental training on FM detection rescued adult abilities for animals raised with moderate hearing loss.

Finally, we asked whether training on the FM detection task in adulthood could improve performance of NH animals when subsequently tested again. NH adults with prior adult training on the FM detection task displayed better detection thresholds (1.14 ± 0.12 log_10_Hz) than NH adults (Wilcoxon test: χ^2^ = 4.77, *df* = 1, *p* < 0.05), untrained CHL adults (Wilcoxon test: χ^2^ = 12.8, *df* = 1, *p* < 0.001), and the juvenile trained CHL adults (Wilcoxon test: χ^2^ = 10.1, *df* = 1, *p* < 0.01). Therefore, the effect of juvenile training on animals with CHL did not restore the highest level of performance that was observed in NH animals.

**Figure 3:**
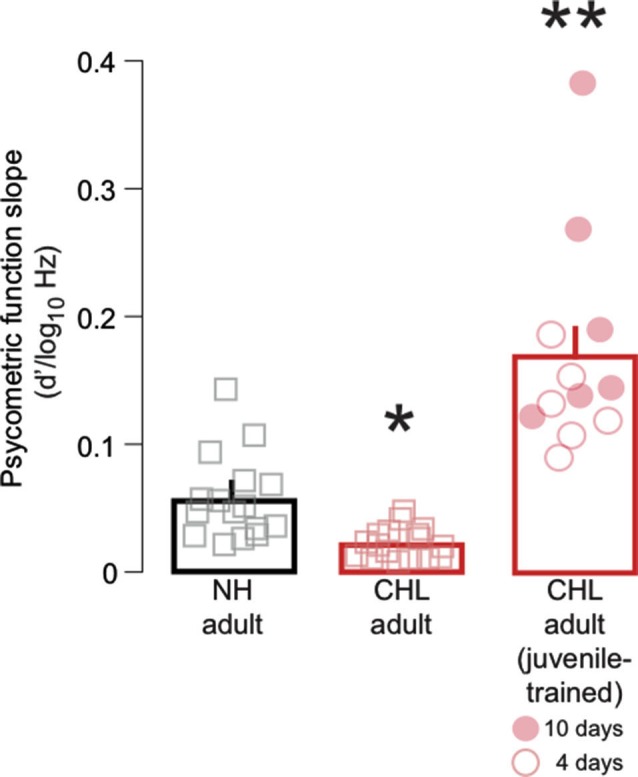
**Training on FM detection as a juvenile improved psychometric function slopes in adulthood**. Average slope of fitted psychometric functions (*d*′/log_10_Hz; mean ± SEM) are plotted for NH adult (black bar, gray squares), CHL adult (red bar; red squares), and CHL adult animals trained on the FM task as juveniles (red bar; red circles). Data points from individual animals are the average of the best 3 days of performance. For the trained animals, open circles indicate 4 days of perceptual training, and closed circles indicate 10 days of perceptual training. Significant differences for between-group comparisons are presented in the text and indicated here by asterisks (** significantly greater than all other groups; * significantly smaller than all other groups).

To determine whether developmental training modifies the variance of performance, the psychometric function slopes were obtained at *d*′ = 1 for the three best sessions. Figure 3 shows a main effect of treatment group (Kruskal-Wallis: χ^2^ = 34.3, *df* = 2, *p* < 0.0001). The average slope for NH adults (0.06 ± 0.01 *d*′/log_10_Hz) was significantly steeper than found in CHL adults (0.02 ± 0.007 log_10_Hz) (Wilcoxon test: χ^2^ = 18.65, *df* = 1, *p* < 0.0001). However, the average slope for juvenile trained CHL-reared animals (0.17 ± 0.02 *d*′/log_10_Hz) was significantly steeper than CHL adults (Wilcoxon test: χ^2^ = 20.4, *df* = 1, *p* < 0.0001), and also significantly steeper from NH animals (Wilcoxon test: χ^2^ = 15.9, *df* = 1, *p* < 0.001). The CHL animals that had been trained for 10 days (Figure 3, closed red circles; 0.2 ± 0.03 *d*′/log_10_Hz) generally displayed steeper slopes than those animals trained for only 4 days (Figure 3, open circles; 0.13 ± 0.03 *d*′/log_10_Hz). This suggests that developmental training on FM detection decreases performance variability for animals raised with hearing loss.

## Discussion

Auditory deprivation during development can induce long-term changes to the central nervous system, and these are associated with perceptual deficits in adulthood (Sanes and Bao, 2009; Sanes and Woolley, 2011). For example, CHL that is induced prior to hearing onset results in poorer performance on both frequency and amplitude modulation detection tasks, even when audibility is compensated (Rosen et al., 2012; Buran et al., 2014), where our lab’s prior work on FM detection in animals with hearing loss tested the theory that there is a critical period for hearing loss on adult perception (Buran et al., 2014). However, the developmental period also provides an opportune time to train animals on perceptual tasks. A brief period of auditory perceptual training during the juvenile period results in a long-term benefit to adult performance on the trained task (Sarro and Sanes, 2011). This current study was a logical extension of this because it led us to explore whether long-term deficits on auditory perceptual skills on FM detection could be rescued with a brief period of training during development. Here, we trained juvenile gerbils with CHL on a FM detection task, and found that their performance on this task in adulthood was better than untrained CHL animals.

The performance of CHL animals was initially assessed immediately at the termination of the training period while animals were still juveniles. At this time, the FM detection thresholds of CHL animals were similar to those displayed by NH juvenile animals (Figure 1). This suggests that the deficits in performance, as measured in adults (Buran et al., 2014), emerge gradually as the duration of deprivation accumulates. Because of inadequate sensory input, the animals with CHL may not display a normal trajectory of improvement. A similar phenomenon is found in children with language based learning disorders who display slower auditory perceptual maturation, and do not attain adult levels of performance (Wright and Zecker, 2004). Consistent with this, children with hearing impairments display immature measures of cortical function when compared to NH children that is only partially restored by cochlear implants, suggesting a delay in the developmental process that does not resolve following an extended duration of hearing (Ponton and Eggermont, 2001).

The results from the present study demonstrate that a brief period of FM detection training during this period is sufficient to improve adult detection FM thresholds in animals with CHL, and thus possibly impede the accumulation of the deficit as a function of age. The two training regimens, 4 or 10 days, were not sufficient to restore a normal level of performance (Figure 2). However, our results indicate that the amount of developmental training can impact adult performance. Thus, those adults that had received 10 days of developmental training had slightly better adult detection thresholds than those with only 4 days of training (Figure 2). The impact of juvenile training on psychometric function slopes (Figure 3) suggests that the limited amount of training given to the juveniles with CHL allowed these animals to become more consistent within a narrower range of FM depths. It is possible that, with more training on the FM detection task during development, these animals may have become more confident across a broader range of FM depths and eventually approached NH adult thresholds, while also modifying the slope closer to NH adults. Taken together, these findings suggest that perceptual learning that occurs during development can provide long-term benefits on perceptual abilities and may, in fact, be able to rescue deficits in perception.

The long-term enhancements to performance were the result of only a brief period of developmental training. Each daily session during development occurred over a period of about 10–15 min in duration and only for a total or either 4 or 10 sessions. Following this training period, animals were not exposed to the training stimuli until reaching adulthood. This suggests that the practice required to improve performance may be small, at least for this task. In fact, the amount of perceptual training required to induce learning does differ, depending on the specific percept being trained (Wright and Fitzgerald, 2001; Wright and Sabin, 2007; Fitzgerald and Wright, 2011). For example, in adult humans, the number of sessions required to show dramatic improvement can be as low as one session for tasks such as amplitude modulation detection and interaural time difference detection, but as high as three sessions for interaural level difference detection (Wright and Fitzgerald, 2001; Fitzgerald and Wright, 2011). In the present study, the amount of training had an impact on the degree of improvement in adulthood (Figure 2), indicating that a further increase of practice could lead to even greater improvements (Hussain et al., 2009), and possibly full recovery of normal adult perception.

### Developmental training may compensate for central impairments

One basis for the ameliorative influence of juvenile training is that central impairments caused by CHL (Xu et al., 2007, 2010; Takesian et al., 2010, 2012, 2013) were either corrected or compensated for by learning learning-associated plasticity. Prior studies demonstrate that primary auditory cortex displays induced modifications for both frequency representation and cortical temporal processing (Recanzone et al., 1993; Weinberger and Bakin, 1998; Beitel et al., 2003; Bao et al., 2004). Moreover, behavioral training has been shown to restore impaired cortical processing in animals with developmentally induced hearing loss. For example, in prelingually deafened cats, behaviorally relevant training leads to enhanced temporal processing in auditory cortex that is close to normal levels (Beitel et al., 2011; Vollmer and Beitel, 2011). Consistent with this, congenitally deaf humans who receive cochlear prostheses early in life display improvements in speech perception and language skills, suggesting a long-term effect of exposure and use of auditory input (Busby et al., 1991; Dawson et al., 1992; Svirsky et al., 2004). Thus, modifications and improvements to an impairment of cortical function may accompany the behavioral improvements displayed by the CHL animals with developmental training.

## Conflict of interest statement

The authors declare that the research was conducted in the absence of any commercial or financial relationships that could be construed as a potential conflict of interest.
